# A Model of Self-Organizing Head-Centered Visual Responses in Primate Parietal Areas

**DOI:** 10.1371/journal.pone.0081406

**Published:** 2013-12-03

**Authors:** Bedeho M. W. Mender, Simon M. Stringer

**Affiliations:** Department of Experimental Psychology, University of Oxford, Oxford, United Kingdom; University of Sheffield, United Kingdom

## Abstract

We present a hypothesis for how head-centered visual representations in primate parietal areas could self-organize through visually-guided learning, and test this hypothesis using a neural network model. The model consists of a competitive output layer of neurons that receives afferent synaptic connections from a population of input neurons with eye position gain modulated retinal receptive fields. The synaptic connections in the model are trained with an associative *trace* learning rule which has the effect of encouraging output neurons to learn to respond to subsets of input patterns that tend to occur close together in time. This network architecture and synaptic learning rule is hypothesized to promote the development of head-centered output neurons during periods of time when the head remains fixed while the eyes move. This hypothesis is demonstrated to be feasible, and each of the core model components described is tested and found to be individually necessary for successful self-organization.

## Introduction

The most familiar reference frame in primate visual neuroscience is the eye-centered reference frame, where the receptive fields of visual neurons are anchored to particular locations on the retina. Another reference frame for visual responses, often studied in the context of visually guided action or behaviour, is the head-centered reference frame. Receptive fields in this reference frame stay in register with the head. Hence mapping of such visual receptive fields at different gaze angles produces responses which are selective for visual targets at particular head-centered locations regardless of the retinal location.

Head-centered visual representations have been thought to potentially play a role in at least three different primate behaviours. First, head-centered visual representations may support sensorimotor integration, where sensory receptors, for example in the retina, cochlea or soma, respond in different reference frames to the motor signal with which they are integrated, and to the motor effectors they may drive. For example, to reach out and grasp a cup of coffee in the periphery of the visual field while reading a newspaper requires the transformation of an eye-centered visual signal into a representation suitable for guiding limbs and posture [1]. The head-centered representation of visual space has been suggested as a possible intermediate representation in such a transformation [2]. Second, head-centered visual representations may contribute to the apparent stability of visual perception in spite of frequent and rapid eye movements, also known as spatial constancy. This stability may be supported by head-centered representations because they encode visual space in a more stable supraretinal reference frame [3]. Third, head-centered visual representations may help to support the execution of spatially accurate saccades, in particular double step saccades [4]. In such a task, two sequential memory guided saccades are made to two distinct locations of recently flashed visual targets. The readily available eye-centered trace activity of the second target cannot guide the second saccade because it does not account for the execution of the first saccade, however a head-centered representation would on the other hand preserve trace activity of the second target in a reference frame invariant to the first saccade [5].

The majority of relevant physiological work has identified a population encoding of head-centered space in the form of eye-centered visual representations with eye position gain modulation. The influence of gaze on area 7a neurons was first established by [6], and the precise interaction between the visual and eye position signals was characterized by [7]. These effects were later also identified in the lateral intraparietal area (LIP) [8]. This work described such gain modulated responses as a multiplicative interaction between a Gaussian retinotopic receptive field and a planar eye position modulation component. The presence of more peaked eye position gain modulation in the parietal occipital area (PO) has also been observed [9], [10]. Later work has demonstrated the existence of explicit single neuron representations of head-centered space in area PO [11], the ventral intraparietal area (VIP) [12] and area LIP [13]. The issue we investigate in this paper is how such head-centered single neuron responses could develop.

A highly influential early model of head-centered neural responses and eye position gain modulation was developed by [14]. This model showed how a neural network trained on independent visual and eye position signals could develop head-centered output units, and eye-centered visual units with planar gain modulation in the hidden layer. This work relied on backpropagation learning [15], which is not a biologically plausible self-organization mechanism. In later work [16] were able to demonstrate similar results without using backpropagation learning. However, this improvement still relied on a form of supervised global error correction learning, which depended on an error term which is unlikely to be present in the cortex. Another model of sensorimotor transformation by [17] also employed a supervised delta rule to modify the synaptic weights [18]. So supervised error-correction learning has been for some time a dominant modelling approach in understanding sensorimotor integration, and the development of head-centered representations in particular.

The model presented in this paper is distinguished from previously published work in that it utilizes a biologically plausible process of visually guided learning to self-organize the synaptic weights and thereby endow the output neurons with head-centered responses. This learning mechanism relies on a combination of a biologically plausible neural network architecture and local synaptic learning rule, combined with plausible assumptions about the natural movements of the eyes and head observed in primates [19].

### Hypothesis

It was hypothesised that the following four core model components would permit head centered visual representations to develop through a biologically plausible process of visually guided learning:

- There is a population of input neurons that encode both the retinotopic location of visual targets and eye position at the single neuron level through coupled visual and eye position receptive fields.
- There is a population of output neurons that compete with each other through mutual inhibitory interactions mediated by inhibitory interneurons.
- The feedforward synaptic connections between the input and output neurons are modified by a local synaptic trace learning rule that encourages individual output neurons to learn to respond to subsets of input patterns that tend to occur close together in time.
- During natural self-motion, there are periods of time when the eyes are moving in the head while the head remains stationary with respect to the visual environment and visual objects also remain stationary within the environment.

Retinotopic visual neurons with eye position gain modulation, satisfying the first premise, have been identified in multiple primate cortical areas [7]–[9]. Premise two is a standard feature of cortical architecture, in which competitive interactions between excitatory neurons are mediated by inhibitory interneurons [20].

Premises three and four are related, in that the latter is the ecological constraint providing the temporal structure which the former exploits.

Under the assumption that visual stimuli are relatively static in a world reference frame, a primate will more often adjust its gaze by moving its eyes rather than the head itself [19]. This behavioural strategy is preferable to making frequent energetically costly and slow head movements to adjust gaze. Evidence for this has been found during exploration of natural environments with free eye, head and body movements [21]. It was found that when there was movement, isolated eye and isolated head movements occurred 33.1% and 13.3% of the time respectively, while the remaining time involved a mixture of movements. These experimental results confirm that the eyes move more frequently than the head. A consequence of this is that, during natural movement, there are periods when the eyes are moving while the head remains stationary with respect to the visual environment. This temporal structure can be exploited by a trace learning rule as follows.

A trace learning rule is a local associative learning rule that incorporates an exponentially decaying temporal trace of past neuronal activity. The effect of such a learning rule is to encourage individual postsynaptic neurons to learn to respond to subsets of input patterns that tend to occur close together in time [22], [23].

If the eyes are moving around a scene containing a visual target while the head remains stationary, then the visual system will receive a sequence of input patterns corresponding to the visual target in different retinal locations but the same relative position with respect to the head. That is, during this period, the visual target will change position in the eye-centered space but it will remain stationary in the head-centered space. The sequence of eye positions and resulting retinal locations of the visual target will be represented by the retinotopic eye-position gain modulated input neurons. The synaptic trace learning rule will bind these input patterns onto the same output representation precisely because these input patterns frequently follow each other in time.

The visual system may be exposed on separate occasions to a number of such input pattern sequences of the visual target in the same head-centered location, but situated in many different retinal locations due to rapid movement of the eyes. Each of these sequences might represent the visual target in a different randomised subset of retinal locations. These randomised sequences would randomly overlap with each other, which would ensure that all possible patterns, corresponding to the same head-centered location but different retinal locations, are brought into temporal proximity with each other. In this way, all of the patterns corresponding to a single head-centered location but different retinal locations would tend to occur clustered together in time. This kind of randomised mixing of input patterns has previously been found to facilitate the temporal binding performed by trace learning [23]. Given this extensive training, a trace learning rule would eventually encourage a subset of postsynaptic output cells to learn to respond to this complete set of input patterns and thereby learn to respond to the visual target at a particular position in the head-centered frame of reference regardless of where the visual target occurs on the retina.

Occasionally the position of the head, itself, will be readjusted, whereupon this process continues with the visual target in a different position in head-centered space. That is, the location of the visual target would be shifted to new head-centered locations by the natural head movements that occur between sequences of rapid eye movements. The learning process could thus be repeated with the visual target presented in many different head-centered locations. Due to the competitive interactions between the output cells, a new subset of postsynaptic output cells would learn to respond to the visual target in each different head-centered location. In this manner, the output layer would eventually develop neurons that cover the entire space of head-centered locations.

## Methods

### Network Architecture

**Figure 1:**
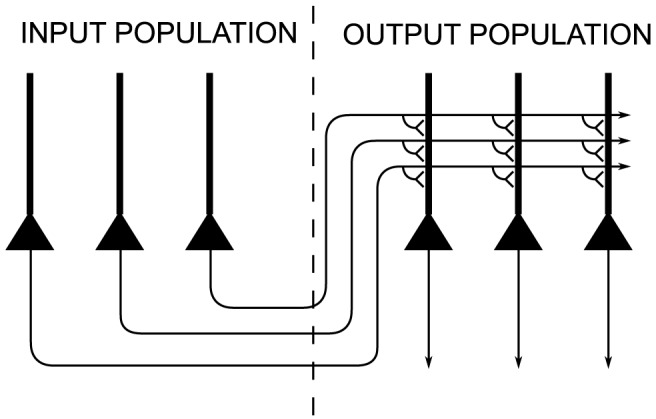
Network architecture. Architecture of 2-layer neural network model. The layer of input neurons on the left are projecting to the competitive output layer on the right. During learning, the strengths of the feedforward synaptic connections from the input layer to the output layer are modified by a trace learning rule.

The architecture of the neural network model is shown in Fig. 1. The network consisted of two layers of neurons, one projecting to the other.

The first layer was a population of input neurons that simultaneously encoded the eye position of the agent and the retinal location of the visual target. These were modelled as retinotopic neurons with eye position gain fields. The retinal and eye position spaces, representing the range of retinal locations and eye positions in orbit, covered 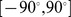 and 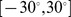 respectively. Neurons in the input layer sent feedforward synaptic connections to neurons in the second layer.

The second layer was a competitive population of 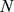 output neurons that competed to represent patterns in the input layer [20]. Neurons in the output layer all received the same number of afferent connections from the input layer, that is 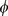 percent of the input population, but each output neuron received connections from its own randomly assigned subset of the input neurons. Neither the input layer nor output layer were topographically organized, however the input layer is presented topographically for visualization purposes. There is no evidence for topography among gain modulated input neurons in either 7a, LIP or PO, nor among head-centered output neurons in the latter two areas.

At the start of each simulation, the strengths of the feedforward synaptic connections from the input layer to the output layer were initialised to random weights in the interval 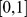. Then the synaptic weight vector of each output neuron was renormalized as is typical in competitive networks [20].

### Training the Network on a Combination of Visual and Eye Position Signals

The network was trained on inputs representing a combination of a visual signal and eye position signal. The visual signal represented the retinal location of a visual target in the scene, and the eye position signal represented the position of the eyes in orbit.

In order to reduce edge effects due to clipping of the input representations, the retinal locations of visual targets were kept within the interval 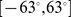, while the position of the eyes was kept within 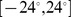.

In each experiment, 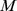 evenly spaced head-centered locations in 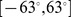 were first chosen. Confining visual targets within this interval of head-centered space ensured that the visual targets always remained in view as the eyes moved, given the eye position space chosen above. Each training epoch was divided into 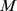 periods, each corresponding to one of the chosen head-centered locations. During such a period, a visual target was located in the given head-centered location while the eyes saccaded through a random sequence of 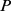 different eye positions uniformly sampled from 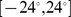. The duration of each fixation was set to 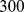
ms, and the saccades between successive eye positions were a constant velocity of 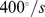.

Thus, during training, the network was presented with sequences of combined visual and eye position input signals that represented the visual targets remaining in fixed head-centered locations while the eyes shifted through randomised positions in the orbit. This was precisley the kind of spatiotemporal structure to the input stimuli required by the self-organization hypothesis.

### Testing the Network

After training, the model was tested by recording the responses of the output neurons for all combinations of 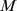 different eye fixation positions and 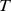 head-centered visual target locations. The data from this testing was used to analyse the receptive field properties of the neurons, including the reference frame of response, receptive field size and receptive field location. In order to test the ability of the model to generalise to new input patterns after training, the responses of the output neurons were tested with combinations of eye position and visual target location that were different to what the model had been trained on. Specifically, the model was tested by having it fixate in 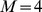 eye positions 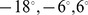 and 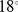, during which a single visual target was placed in each of 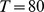 head-centered target locations within 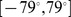 in increments of 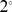. For each combination of eye position and head-centered visual target location the model fixated for 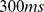, and the firing rates of all neurons in the output layer were saved at the end of this period for analysis. During testing there was no learning and all model variables were reset between different combinations of eye position and head-centered visual target location. Therefore there is no order effect in the testing.

### Neuronal and Synaptic Dynamics

#### Input Layer

The neurons in the input layer were modelled by imposing a firing rate function that simulated the response properties of retinotopic neurons that were modulated by eye position gain fields. Such neurons have been found in a number of areas of the primate brain, including area PO [9], 7a and LIP [8].

The response function mapped the eye position, denoted by 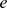, and the retinal location of a single visual target, denoted by 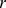, onto the instantaneous firing rate of the 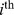 input neuron, denoted by 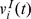, within the range 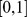. Specifically, the response was described by
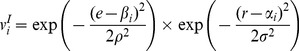
(1)


This response function was composed of a product of two components: the first component represented the eye position gain, while the second component represented the retinotopic location of the visual target in the scene.

The parameter 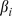 represented the preferred eye position for the 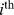 input neuron, with the width of the corresponding Gaussian eye position tuning curve determined by the standard deviation 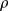. The parameter 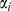 specified the preferred retinal location of a target stimulus for the 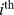 input neuron, and the standard deviation 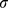 determined the width of the corresponding Gaussian retinal tuning curve.

This sort of gain field, referred to as a ‘peaked’ gain field, has been reported in area PO [9]. Each input neuron was set to respond maximally to a unique combination of retinal target location 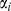 and eye position 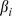. The population of input neurons covered the entire two dimensional space resulting from combinations of eye position and retinal target location in integer steps of 1 degree in each dimension.

#### Output Layer

For the 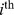 neuron in the competitive output layer there were three dynamical quantities defined: a trace value 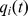, an internal activation 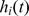 and an instantaneous firing rate 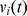 [24].

The activation was governed by the equation
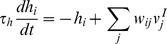
(2)where 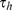 was a time constant common for all neurons in the output layer and 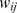 was the synaptic weight of the synapse from the 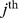 input neuron to the 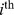 output neuron.

The firing rate was given by the equation
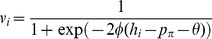
(3)where 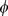 was the sigmoid slope and 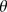 was the threshold. The parameter 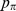 was used to regulate the level of competition between neurons in the output layer, and thereby control the proportion of neurons that remained active. Specifically, 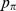 was set to the activation value at the 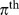 percentile point of the distribution of neuronal activations within the output layer. For example, if 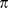 was set to 90, then 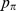 was set to the top tenth percentile activation value. This was a practical means of implementing competition within the competitive output layer, which in cortex is implemented via inhibitory interneurons [24]. This way of implementing competition has been previously used in competitive neural network models of the primate visual system with trace learning [25].

#### Trace Learning

Trace learning rules utilize a temporal trace of recent neuronal activity in order to encourage postsynaptic neurons to bind together subsets of input patterns that occur close together in time. The trace value for the 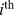 neuron in the output layer was denoted by 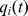 and was governed by the equation
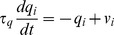
(4)where 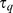 was a time constant common for all neurons in the output layer.

During training the strength of the synapse from the 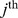 input neuron to the 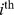 output neuron was governed by the trace learning rule
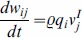
(5)where 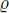 was the learning rate, 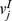 was the firing rate of the 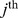 input neuron and 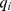 was the trace value of the 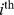 output neuron.

Finally, to prevent unbounded growth of the synaptic weights during training, the length of the weight vector for each output neuron 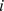 at time 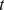, that is 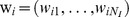 where there are 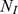 input neurons, was renormalized by setting
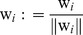
(6)after each weight update [24]. Experimental evidence for renormalisation of synaptic weights in the brain has been provided by [26].

### Simulation of the Differential Model

The coupled differential equations 2, 4 and 5 were integrated numerically using the Forward-Euler scheme, where the numerical time step 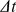 was set to one tenth of the neuronal time constant 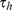. For all simulations the stability of the results was manually confirmed by checking that the qualitative nature of the results remained invariant at the single neuron level over reductions in time step and increases in the number of training epochs.

The combined visual and eye-position input signals during training and testing were simulated dynamically and sampled at 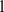
kHz. Then, where necessary, linear interpolation was used to compute the numerical inputs to the discretized Forward Euler model equations, which required input values at every numerical time step 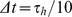.

### Analysis of Network Performance

Let 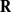 be a matrix containing the responses of a given neuron during testing, where 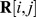 denotes the firing rate when the model was fixating in the 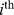 eye position 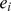 and the visual target was in the 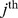 head-centered location 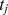, as recorded during the testing protocol described above. The vector 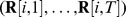 is referred to as the response vector at the 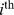 eye position. The number of eye positions during testing is denoted by 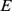, while the number of head-centered locations for visual targets during testing is denoted by 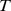. The indexing of eye positions and head-centered target locations were ordered from left (negative) to right (positive), that is 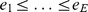 and 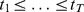.

#### Reference Frames

To determine which reference frame an output neuron was responding in during testing, two separate metrics were applied that reflected to what degree the neuronal response was compatible with either an eye-centered or head-centered reference frame, and then the values of these two metrics were compared.

The head-centeredness metric computed the degree to which the head-centered response vectors of a neuron remained stable across different eye positions. The head-centeredness metric measured the degree of such stability for a given output neuron by averaging correlations between response vectors for different eye positions, that is
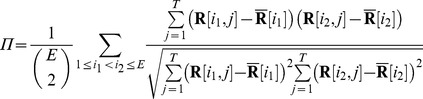
(7)where
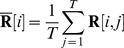
(8)


This yielded a metric which was referred to as the *head-centeredness* of the output neuron, and it was bounded between 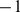 and 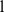, where a perfect correlation of 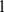 indicated a perfectly head-centered response.

A very similar analysis was done to quantify the compatibility of the responses of the output neuron with an eye-centered frame of reference. That is, a visual neuron is judged to respond in an eye-centered frame of reference to the extent that its eye-centered response vectors remain stable across different eye positions. The eye-centered analysis proceeded as follows. To reiterate, each response vector 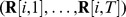 was the result of testing over the same set of head-centered locations, but with the model fixated in a distinct eye position. Therefore, each response vector also corresponded to a unique range of retinal locations. The *intersection* of these retinal ranges corresponded to different portions of each response vector, and it was these portions that were subject to correlation analysis. Specifically, 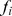 denotes the first vector position in the 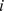 th response vector to be included, and the 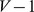 next positions are included as well such that the subvector 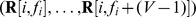 is the vector being used for the correlation analysis. The derivation of 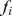 and 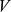 are found in the Appendeix S1. This gave the metric
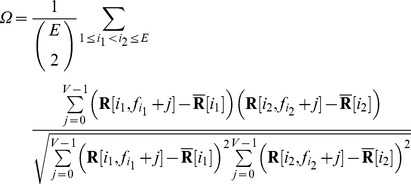
(9)where
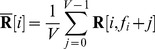
(10)


This was referred to as the *eye-centeredness* of the output neuron, and it was bounded between 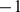 and 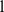, where a perfect correlation of 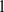 indicated a perfectly eye-centered response. Response vectors which had no response for the extracted ranges were excluded from the correlation, and a neuron without a response within this range of retinal locations at any eye position was excluded from further analysis.

Both of the reference frame metrics were finally combined into a receptive field index (RFI) which classified each neuron along a spectrum from eye-centered to head-centered. The RFI was defined as
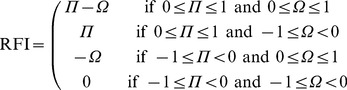
(11)


The index was a continuous valued function bounded between 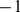 and 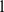. An output neuron with either a positive, negative or nil RFI value was classified as head-centered, eye-centered or undetermined respectively. In general, a large positive value for the RFI indicated better compatibility with a head-centered reference frame than a smaller positive value. Similarly, a large negative value for the RFI indicated better compatibility with an eye-centered reference frame than a smaller negative value.

#### Receptive Field Location

The head-centered receptive field location of an output neuron was determined as follows. First, the head-centered receptive field location at each eye position 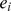 for 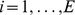 during testing was computed. The head centered receptive field location for the 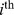 eye position was computed using the centre of mass of the head-centered response vector at this eye position. Next, the average of these head-centered locations over all eye positions was computed, and this averaging is shown to be optimal in the Appendix S2. This gave the final metric for each neuron
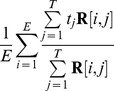
(12)


#### Coverage

It was important to assess how the receptive fields of head-centered output neurons were distributed over the range of 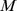 head-centered locations used during training. This was to establish whether the space of head-centered locations was evenly represented by differently tuned output neurons, and in particular whether each training location was preferred by at least one head-centered output neuron. In this analysis it was then determined how the receptive field locations of head-centered output neurons were distributed among the head-centered locations where visual targets were presented during training, denoted by 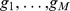.

This distribution was determined by first assigning each head-centered neuron to the closest head-centered training location. Let 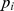 denote the fraction of head-centered neurons assigned to training location 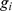. To ensure that all locations had at least one neuron assigned to them, and also quantify the extent to which all locations were evenly represented, the normalized entropy of this distribution was computed by
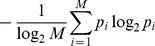
(13)and this was referred to as the *coverage* of the model. A perfectly uniform distribution would give a maximal value of 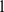, and if there was some 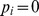 it was undefined and there was said to be no coverage.

#### Receptive Field Size

To quantify the receptive field size of a visual neuron, a firing rate threshold is often chosen to demarcate the responsive region or receptive field. In the following analysis, a neuron was considered responsive when it was firing above a threshold rate 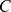 no less than 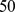
% of its maximal rate across all eye positions and head-centered visual locations, that is
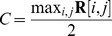
(14)


For each eye position, this threshold was used to isolate the responsive head-centered regions of the given neuron. The total size of all such regions was added up, estimating the total receptive field size at the given eye position. The final receptive field size of the given neuron was the average of all these estimates from the different eye positions.

To isolate the responsive regions of a neuron at the 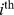 eye position, a head-centered piecewise linear response function 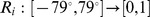 was derived from the 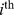 response vector using linear interpolation. Responsive regions were isolated by first finding all solutions 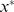 to the equation 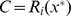, and then identifying the regions of head-centered space where the interpolated neuronal response was above threshold, denoted by 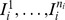. In practice there was hardly ever more than a single responsive region per eye position 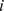, that is 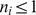. Finally, the total size of all responsive regions 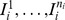 for each eye position was averaged across all eye positions, that is
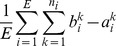
(15)where 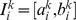 was the 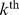 region at the 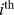 eye position, of which there were 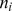 in total. On a few rare occasions, a head centered neuron did not actually respond in one of the eye positions. In this case, that eye position was excluded from the averaging procedure carried out in equation 15.

## Results

### Self-Organizing Model

**Figure 2:**
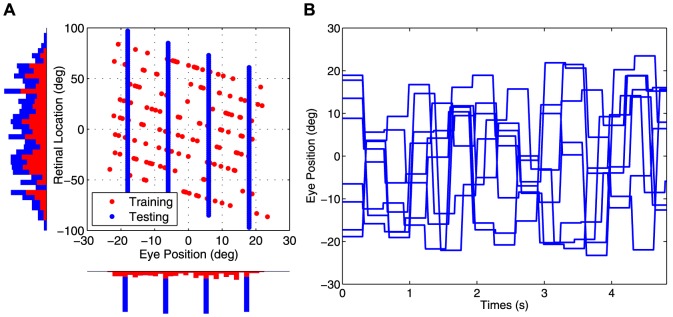
Stimuli data points. Simulated movements of the eyes and head-centered locations of visual targets during training and testing. (**A**) Scatter plot in which each point corresponds to a single fixation during either training (red) or testing (blue). The fixation points are plotted as a function of the eye position (abscissa) and the retinal location of the visual target (ordinate). Each of the diagonal lines of red points corresponds to a period during training when the visual target was fixed in one of the eight head-centered target locations while the eyes moved. The vertical lines of blue points correspond to the four eye positions in which the network was tested. (**B**) Multiple plots showing how the eye position is shifted through time in a randomised manner during training. Each plot corresponds to a different period during which the visual target is maintained in a fixed head centered location.

This experiment explored the feasibility of the self-organization hypothesis presented in this paper. The model had 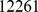 neurons in the input population, and 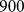 neurons in the output layer. Each output neuron received 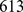 afferent synaptic connections from a randomly assigned subpopulation of the input population. At the beginning of training, the synaptic weights were set to random values. Then the synaptic weight vector of each of the output neurons was renormalized according to equation 6. The network was then trained for 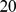 epochs. During each training epoch, a visual target was presented for approximately 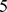
s in each of the eight head centered training locations: 
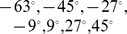
 and 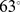. For each period where the visual target was in a fixed head centered target location, the eye position was varied continuously through time as the model made a series of saccades and fixations. During each such period, the model performed 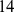 saccades interleaved with 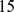 fixations, where each fixation lasted 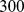
ms. Each saccade was at a constant velocity of 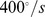, and it was directed to a random eye position within the range 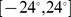. Each training epoch thus lasted for approximately 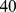
s, and the entire training of the network was completed after about 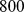
s of simulated time. The model was tested as previously described. Figure 2 shows the simulated movements of the eyes and head centered locations of visual targets during training and testing. The parameters for the model are given in table 1.

**Table 1:** Parameters of self-organizing model.

Parameter	Symbol	Value
Number of target locations	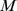	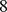
Fixation sequence length	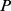	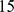
Number of training epochs	-	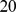
Width of eye position tuning curve	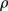	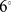
Width of retinal tuning curve	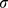	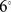
Output neuron population size	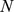	900
Input neuron population size		12261
Trace time constant	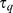	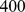 ms
Activation time constant	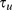	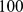 ms
Activation function slope	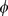	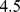
Activation threshold threshold	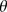	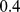
Sparseness percentile	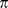	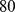 %
Learning rate	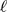	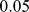
Synaptic connectivity	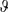	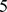 %

Parameters of self-organizing model.

**Figure 3:**
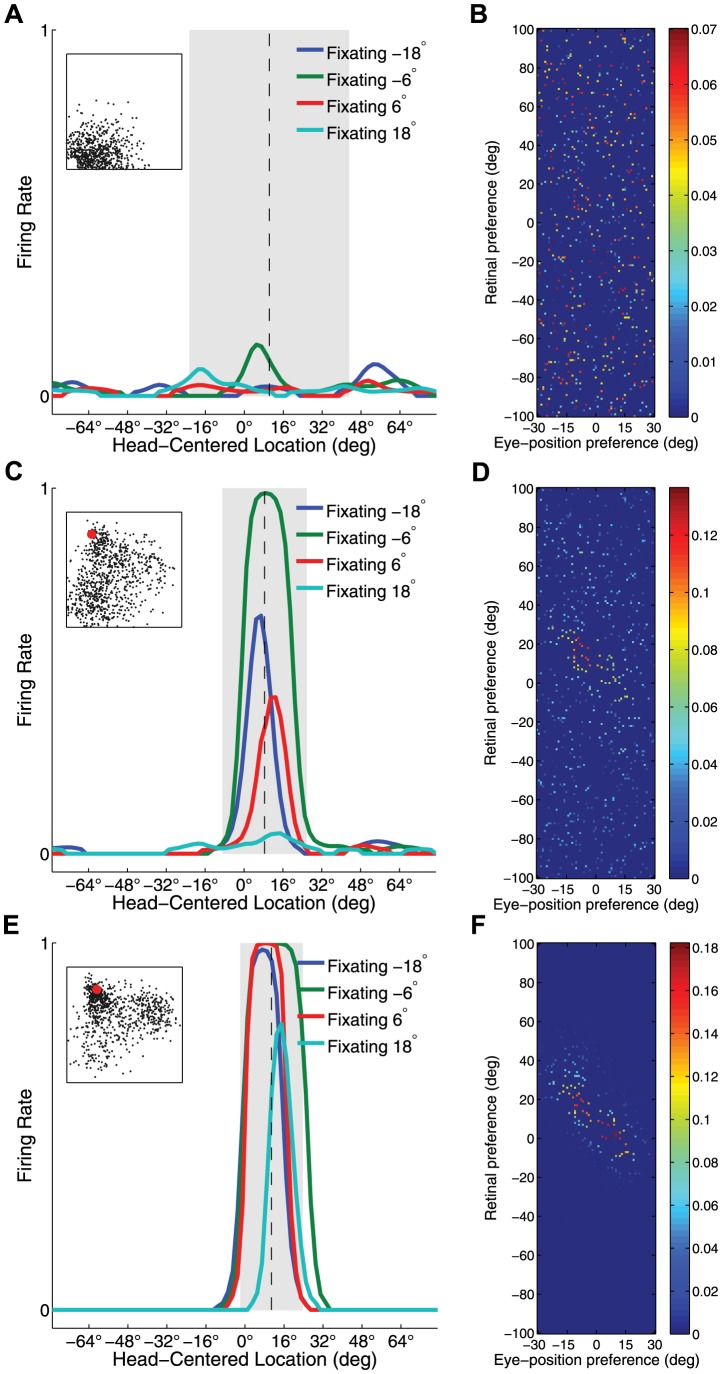
Neuron #79 firing responses and weight vector before and after training. The development of the firing responses and synaptic weights of output neuron #79 before and after training. Results are presented before training (top row), after 10 training epochs (middle row) and after 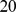 training epochs (bottom row). Plots in the left column show the firing rate responses of neuron #79 during testing. Within each plot, each curve corresponds to a fixed eye position while a visual target is presented in a range of head-centered locations. The vertical line shows the decoded head-centered receptive field location, and the grey bar shows the decoded receptive field size of the neuron. The minature scatter plot shows the response characteristics of all neurons in the output layer, where each neuron is plotted as a point corresponding to that neuron's particular combination of head-centeredness (ordinate) and eye-centeredness (abscissa). The neuron whose firing rate responses have been plotted is shown in the scatter plot by a red mark. Plots in the right column show the synaptic weights of synapses afferent to neuron #79. Within each plot the synapses have been arranged topographically by the effective preference of the input neuron for retinal location 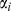 and eye position 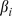.

Figure 3 shows how the firing responses and synaptic weights of one the output neurons #79 develop during successive stages of training.

The responses of the output neuron prior to training exhibited no consistent structure in head-centered space across the different eye positions (Fig. 3a). However, at both 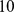 and 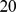 epochs there was a maximal response to the same head-centered location across all four eye positions (Fig. 3c,e), demonstrating more head-centered response characteristics. Before training, the neuron had head-centeredness 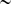
0.12, eye-centeredness 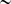-0.2, receptive field location 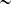
10, and receptive field size 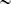
65. However, the corresponding metric values at 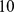 training epochs were 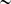
0.79, 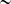
0.13, 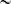
8 and 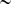
34, indicating the development of head-centered responses. This became more pronounced at 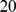 training epochs, where the response profiles became more sharply focussed in the head-centered space and the metric values were 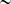
0.78, 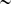
0.18, 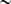
11 and 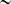
25, respectively.

There was also a correspondance between the weight vector of the output neuron and the response of the neuron during testing at each stage of training (Fig. 3b,d,f). Prior to training the afferent synapses had random values and no structure in terms of the relationship between the weight of a synapse and the characteristics of the presynaptic neuron. However, during training a clear diagonal structure developed in the synaptic weights. The most potentiated synapses were those originating from input population neurons 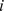 which had a preference for a retinal location 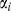 and eye-position 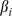 corresponding to the head-centered location which the output neuron preferred. These particular input neurons lay on the diagonal line that is evident in the synaptic weights at 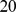 training epochs shown in Fig. 3f. This corresponded to the diagonal line with gradient 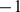 and retinal target location intercept 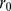 at 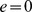 equal to the head-centered location preferred by the neuron.

In summary these results showed that, after 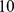 and 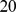 epochs of training, the neuron responded in a head-centered frame of reference, while it did not prior to training. Moreover, at both 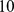 and 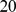 training epochs there was reasonably good agreement about the location of the receptive field in head-centered space according to the neuron's firing rate responses and learned synaptic weights. There was also a degree of eye position modulation evident in the responses of this head-centered neuron.

**Figure 4:**
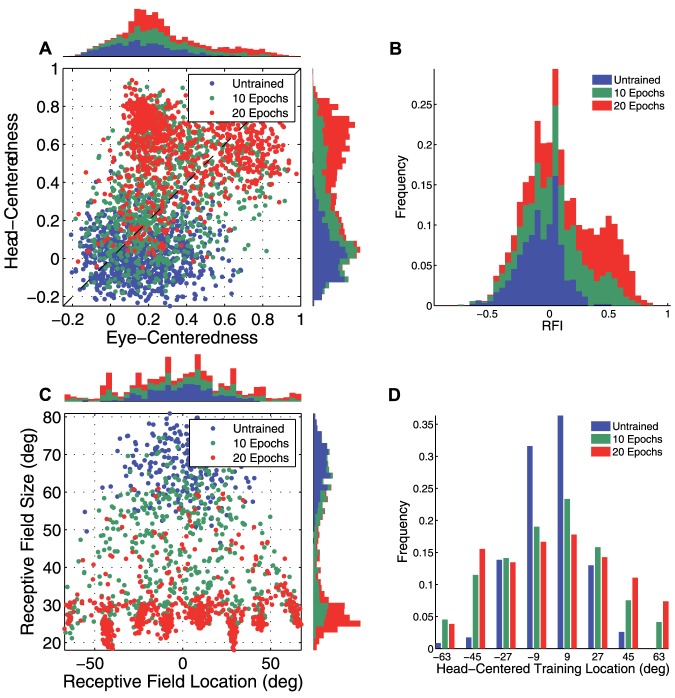
Population analysis of receptive field properties. Population analyses of receptive field properties of output neurons during succcessive stages of training of the self-organizing model. Results are presented before training, after ten training epochs, and after 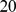 training epochs. (**A**) Scatter plot shows the reference frame response characteristics of all neurons in the output layer, where each neuron is plotted as a point corresponding to that neuron's particular combination of head-centeredness and eye-centeredness. Neurons from the untrained model are shown in blue, neurons from the trained model are shown in red. (**B**) Distributions for receptive field index values before and after training. (**C**) Scatter plot showing the combination of head centered receptive field size and head-centered receptive field location of all head-centered output neurons before and after training. (**D**) Histograms showing the frequency distribution of the numbers of output neurons that responded preferentially to each of the head-centered locations which were used to train the model.

Figure 4 presents the population analyses of the receptive field properties of the output neurons after 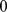, 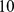 and 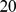 epochs of training, and population statistics are given in table 2. The model before training and after 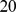 epochs of training is herein referred to as the untrained and trained model respectively.

**Table 2:** Results for self-organization experiment.

	Untrained		10 Epochs		20 Epochs	
	All	RFI 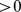 ( 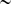 26%)	All	RFI 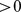 ( 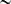 59%)	All	RFI 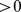 ( 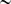 69%)
Head-centeredness	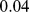 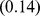	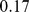 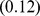	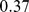 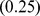	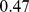 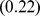	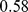 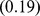	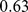 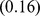
Eye-centeredness	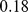 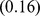	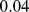 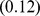	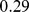 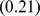	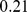 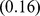	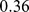 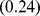	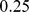 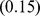
RFI	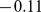 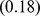	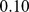 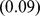	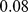 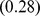	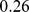 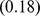	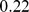 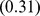	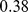 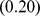
RF Location	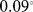 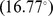	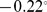 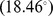	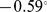 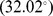	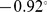 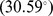	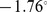 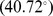	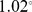 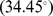
RF Size	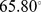 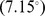	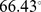 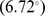	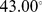 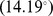	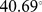 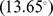	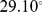 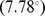	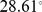 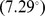

Population summary statistics of response properties of output neurons in the model at three different stages of training. Results for the untrained model are shown in the left two columns, results for the model after 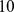 epochs of training are shown in the middle two columns and the results for the model after 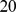 epochs of training are shown in the rightmost two columns. For each stage, results are presented in two subcolumns: statistical measures computed over all output neurons are shown in the left subcolumn, while measures computed over neurons with a receptive field index greater than zero indicating head-centered responses are shown in the right subcolumn. Each row corresponds to a different performance metric: head-centeredness, eye-centeredness, receptive field index, head-centered receptive field location, and head-centered receptive field size. Each cell of the table shows the mean and standard deviation (in parentheses) of the peformance metric over the relevant population of output neurons.

Most output neurons in the untrained model had head-centeredness values and eye-centeredness values clustered close to zero. However, many more neurons in the fully trained model had head-centeredness values clustered close to 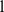, with eye-centeredness values close to zero (Fig 4a). In particular, table 2 confirms that training the network led to an increase in the average head-centeredness over the population of output cells from 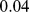 to 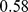. Also, the average RFI was increased from 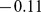 to 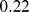 with training. The proportion of neurons with a head-centered response, as indicated by a positive RFI, was increased from 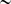
26% to 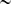
69% during training. Among head-centered neurons the average head-centeredness was increased from 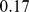 to 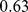 by training. In summary, these results showed that training the network had the effect of increasing the number of head-centered neurons, and also refined the response characteristics of individual neurons to be more compatible with a head-centered frame of reference.

Head-centered neurons in the fully trained model had receptive fields clustered around one of the eight head centered training locations, and there was a similar distribution of receptive field sizes for each of these head centered locations (Fig 4c,d). In contrast, the head-centered neurons in the untrained model had receptive fields covering a more localised central region of head-centered space, and a wider range of receptive field sizes across head-centered space. The average receptive field location was near zero for both the untrained and fully trained models, which indicated that there was no lateralized bias in receptive field locations in these models. However, while the fully trained model had head-centered neurons covering all eight head-centered training locations with a coverage of 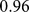, the untrained model had no head-centered neurons for the two most eccentric head centered locations (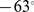 and 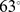). Lastly, the average receptive field size decreased from 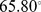 to 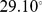 during training. This receptive field size depends on a range of factors, among which are the size of the receptive fields in the input layer, the level of competition in the output population, and the number of training locations during training. Interestingly, as the level of competition (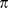) or the number of training locations (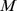) increases, the receptive field size decreases. In both cases, an increase leads to more vigorous competition among output neurons, which in turn more severly truncates the skirts of the receptive field curve of output neurons. Thus, in summary, training the network developed head-centered neurons covering head-centered space, and also increased neuronal selectivity by reducing the sizes of the head centered receptive fields.

**Figure 5:**
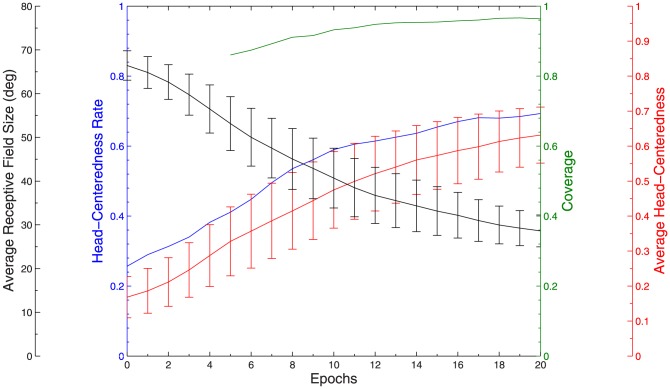
Population analysis for varying number of epochs of training. Population analyses of receptive field properties of output neurons in the self-organizing model during succcessive training epochs. There are four plots as follows. The average receptive field size curve (black) shows the average size of the head centered receptive field among head-centered neurons, and the error bars represent the standard deviations. The head-centeredness rate (blue) was the fraction of output neurons that were head-centered. The coverage curve (green) was the coverage of the head-centered training locations by the output neuron population after the given number of epochs of training, where missing data points before epoch 5 were due to at least one of the eight head-centered training locations not being represented by the output cells. The average head-centeredness curve (red) was the average head-centeredness value among all head-centered neurons, and the error bars were the standard deviations.

The output neurons in the model self-organize their afferent synaptic connections over the course of 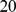 training epochs by visually-guided competitive learning [20]. In such a model, it is important to investigate whether the learning process converges and the response characteristics of the output neurons settle down asymptotically to some form of stable behaviour. The impact of each successive training epoch on the receptive field properties of output neurons was examined by plotting key summary statistics as a function of training epoch in Figure 5. It could be seen that the fraction of output neurons that were head-centered, the average head-centeredness among head-centered neurons, and the coverage of the head centered training locations all increased close to monotonically during training. Also, the average head-centered receptive field size decreased monotonically during training. Most importantly, it was found that these summary statistics converged on steady values after further training. Thus, the key performance characteristics of the model developed close to monotonically in the desired way as the number of training epochs increased.

### Input Neurons with Coupled Visual and Eye Position Receptive Fields

It was hypothesised that the model required input neurons with coupled visual and eye position receptive fields in order to be able to develop head-centered output neurons. In this experiment the necessity of this premise was investigated by exploring how decoupling the visual and eye position components of the receptive fields of input neurons would affect the self-organization of the model. In order to decouple the visual and eye position components, half of the input neurons were set to respond purely to the retinotopic location of a visual target, while the other half of the input neurons were set to respond purely to the eye position. The experiment otherwise had the same parameters as used in previous experiment.

The expected result was that with decoupled receptive fields in the input population the output layer would be unable to form head-centered representations. The reason for this is that the single layer of synapses between the input layer and output layer would not be able to implement a suitable mapping because all of the input neurons would by definition participate in encoding any given location in head-centered space. This problem is solved in a model with input neurons with coupled visual and eye position receptive fields because the individual input neuron has a selective response corresponding to a particular location in the head-centered space.

**Figure 6:**
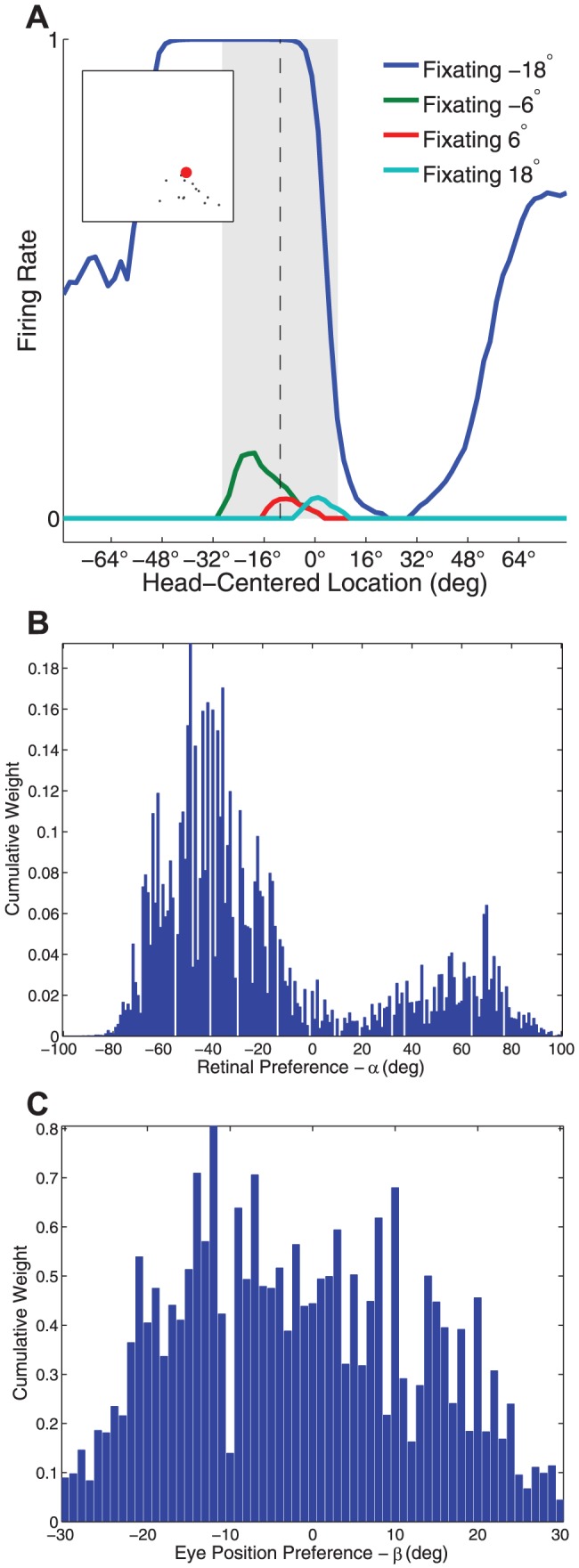
Neuron #170 firing responses and weight vector. Analysis of one of the output neurons #170 from the model with input neurons with decoupled visual and eye position receptive fields. Results are presented after training. (**A**) The firing rate responses of the output neuron. (**B**) and (**C**) Histograms of afferent synaptic weights onto the output neuron from input cells that represent the retinal target location and eye position, respectively. The histograms in (**B**) and (**C**) were produced by finding the sum of all synaptic weights from input neurons with the given retinal location (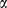) or eye position preference (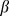) respectively. Both histograms have bin sizes of 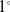.

Table 3 presents typical population summary statistics of the response properties of output neurons from the model with input neurons with decoupled visual and eye position receptive fields. The statistics were computed over subsets of output neurons for which the head-centeredness and eye-centeredness metrics were mathematically defined. This meant that 10% and 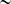
98% of neurons had to be discarded from further analysis in the untrained and trained model respectively. The table shows that the average head-centeredness did increase from 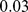 to 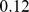 after training. However, the greatest head-centeredness value found among the trained ouput neurons was only 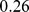. Moreover, all output neurons had a negative RFI, indicating an eye-centered response. Figure 6 presents the firing responses and synaptic weights of one of the output neurons #170 from the model. The results are shown after training. It can be seen that the neither the firing responses of the output neuron nor the afferent synaptic weights were consistent with a head centered receptive field.

**Table 3:** Results for experiment with input neurons with decoupled receptive fields.

	Untrained	Trained
Head-centeredness	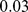 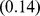	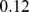 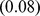
Eye-centeredness	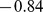 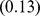	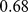 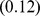
RFI	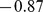 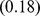	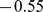 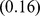
RF Location	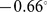 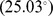	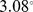 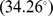
RF Size	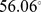 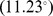	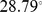 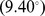

Population summary statistics of response properties of output neurons in the model with decoupling of the visual and eye position components of the receptive fields of input neurons. Results are given before training (left column) and after training (right column). Each row corrresponds to a different performance metric: head-centeredness, eye-centeredness, receptive field index, head-centered receptive field location, and head-centered receptive field size. Each cell of the table shows the mean and standard deviation (in parentheses) of the performance metric over a subset of output neurons described in the text.

The results described here were typical over a broad range of model parameter values. Thus, these results demonstrated that decoupling the visual and eye position receptive fields of input neurons prevented the development of head-centered output neurons during training, and in fact had the opposite effect.

### Competitive Interactions between Output Neurons

It was hypothesised that the model required competitive interactions between the output neurons in order to develop head-centered representations in the output population. The function of such competitive interactions is to ensure that only a small subset of output neurons remain active at any time. The effect of this, when combined with some form of associative synaptic learning, is to encourage individual output neurons to learn to respond highly selectively to distinct subsets of input patterns, with different output neurons learning to respond to different subsets. The subsets of input patterns that the output neurons learn to represent reflect natural groupings within the space of input patterns, and may also depend on the form of associative learning rule used. This kind of learning process is known as *competitive learning* [20].

In this experiment, the necessity of competitive interactions between output neurons was investigated by exploring how turning off these competition interactions affected the self-organization of the model. In previous experiments, the competitive interactions between output neurons were mediated by a dynamically adjusted response threshold 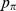 ensuring that all neurons with activation less than the 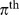 percentile of the activation distribution would fall below the sigmoidal response threshold, as specified in equation 3. In the simulations described next, turning off the competitive interactions between output neurons was achieved by setting the activation threshold to 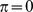. This effectively permitted all of the output neurons to remain active. The experiment otherwise had the same parameters as the first experiment.

**Figure 7:**
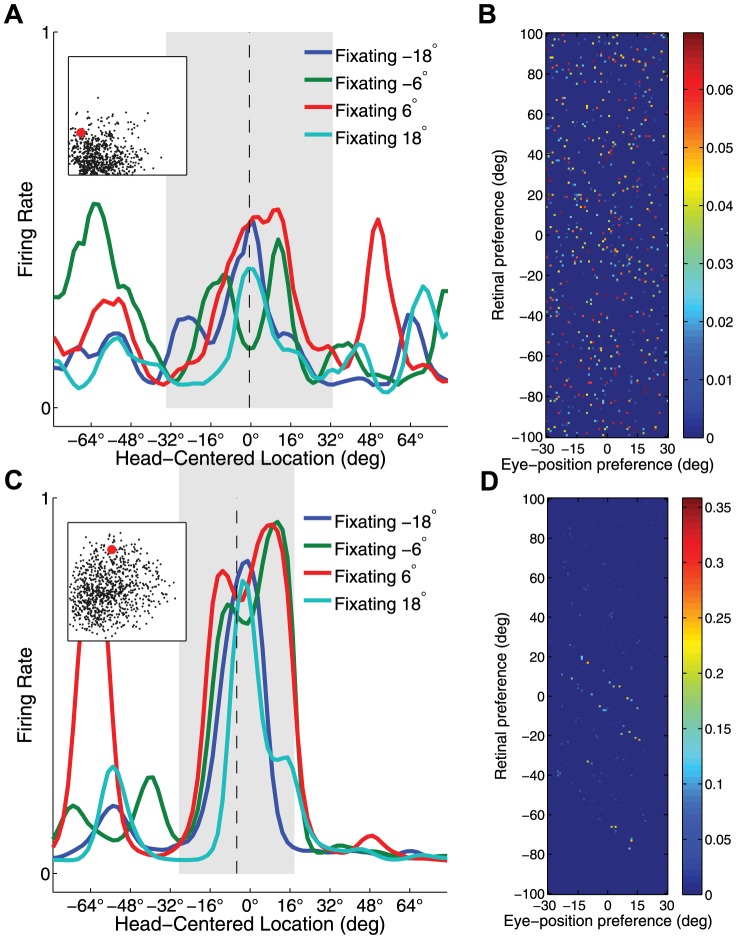
Neuron #409 firing response and weight vector. Results from a simulation without competitive interactions between output neurons. The Figure shows the firing responses and synaptic weights of one the output neurons #409 before training (top row) and after 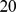 training epochs (bottom row).

Results from a simulation without competitive interactions between output neurons are given in Figure 7, which shows the firing responses and synaptic weights of output neuron #409 before and after training. Prior to training the neuron responded to a large portion of head-centered space at all eye positions (Fig. 7a). Although there was a weak response in the center of head space across all eye positions. This was reflected in head-centeredness and eye-centeredness values of 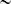
0.30 and 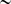
0.015 respectively, indicating a modest head-centered response. The size of the receptive field before training was 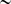
66. After training the responses were more coordinated across all eye positions (Fig. 7c), with a much larger response localised in the middle of the head-centered space. The head-centeredness and eye-centredness values were 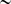
0.75 and 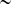
0.30 respectively, showing that training had significantly increased the head-centeredness of the neuron. The weight vector (Fig. 7d) reflected this as well. However, unlike previous simulations with competition in the output layer, the receptive field size of this neuron after training remained very large, approximately 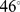. Thus, although the neuron was head-centered, the head-centered receptive field was so large that the neuron would not convey much information about the location of a visual target in the head-centered space. This finding was also typical of other neurons in the output layer after training without competitive interactions between output neurons.

**Figure 8:**
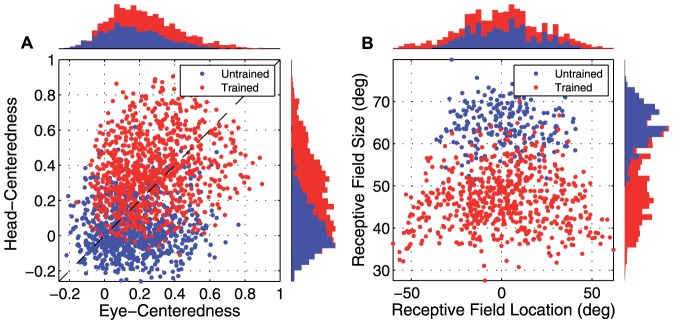
Population analysis in experiment without competition in output layer. Simulation results without competitive interactions between output neurons. Population analyses of the receptive field properties of output neurons are presented before training (blue) and after training (red). (**A**) Scatter plot showing the reference frame response characteristics of all neurons in the output layer. (**B**) Scatter plot showing the combination of head centered receptive field size and head-centered receptive field location of all head-centered output neurons.

Further results from the same simulation are given in Figure 8, which presents the population analyses of the receptive field properties of the output neurons before and after training. Population summary statistics for the simulation are given in table 4. The fraction of output neurons that were head-centered increased from 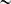
0.25 to 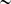
0.67 with training, and among head-centered neurons the average head-centeredness increased from 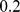 to 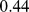. However, unlike previous simulations with competition in the output layer that developed highly selective head centered output neuron responses, the average receptive field size among head-centered neurons had a relatively large value of 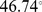. This was 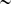
68% larger than the corresponding receptive field size of 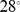 in the experiment described above with competition between output neurons.

**Table 4:** Results for experiment without competition in output layer.

	Untrained		Trained	
	All	RFI 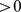 ( 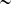 25%)	All	RFI 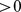 ( 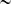 67%)
Head-centeredness	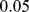 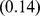	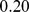 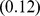	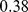 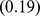	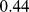 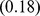
Eye-centeredness	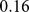 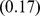	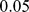 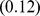	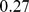 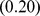	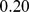 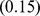
RFI	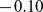 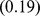	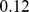 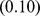	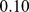 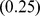	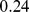 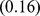
RF Location	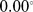 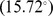	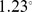 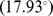	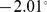 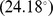	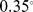 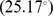
RF Size	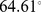 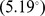	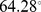 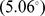	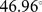 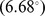	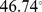 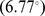

Simulation results without competitive interactions between neurons in the output layer. Population summary statistics of response properties of output neurons are shown before training (left two columns) and after training (right two columns). For each of these two stages of training, statistical measures computed over all output neurons are shown in the left subcolumn, while measures computed over neurons with a receptive field index greater than zero are shown in the right subcolumn.

In summary, these results show that, while the model without competition between output neurons is capable of producing more head-centered output neurons after training, these output neurons actually develop much larger receptive fields in head centered space. This means that such output neurons would in fact convey much less information about the head-centered location of a visual target than output neurons from a model that incorporated competition within its output layer.

### Temporal Binding

It was hypothesised that the model required a synaptic learning rule that incorporated a memory trace of recent neuronal activity in order to encourage output neurons to bind together input patterns that tended to occur close together in time. If, for much of the time, visual targets tend to remain fixed with respect to the head while the eyes move, then such a trace learning rule will encourage individual output neurons to learn to respond when the visual target is in a particular head-centered location regardless of the position of the eyes and hence the retinal location of the target. If the memory trace is removed from the learning rule, then this temporal binding cannot occur which should lead to a failure of the output layer to develop head centered representations.

A memory trace can be incorporated into the synaptic learning rule in a number of alternative ways [27], [28]. Equation 5 gives an example of a learning rule in which an explicit trace term 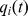 has been incorporated.

However, an alternative, and even simpler approach, is to use a standard hebbian learning rule
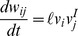
(16)where 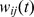 is the synaptic weight from presynaptic neuron 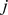 to postsynaptic neuron 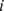, 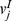 and 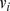 are the firing rates of the pre- and postsynaptic neurons respectively, and 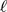 is the learning rate. The hebbian learning rule 16 is combined with synaptic weight normalization 6 to prevent unbounded growth of the synaptic weights during training. If the time constant 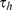 governing the activation of the postsynaptic neuron in equation 2 is increased, then this will lengthen the period of time taken for the activations and hence firing rates of these neurons to decay. In this case, the sustained neuronal activity effectively provides an implicit memory trace in the hebbian learning rule 16 that can promote temporal binding of input patterns that occur close together in time.

The necessity of a memory trace in the synaptic learning rule was investigated in two sets of simulations. In the first set of simulations, the trace rule 5 was tested. Here the duration of the memory trace was varied by varying the time constant 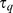 of the neuronal trace 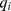 in equation 4 over three orders of magnitude. In the second set of simulations, the Hebbian learning rule 16 was tested. In this case, the duration of the effective memory trace was controlled by varying the neuronal activation time constant 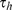 in equation 2 over the same range.

In both sets of simulations, it was expected that decreasing the relevant time constant, 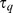 or 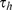, should reduce temporal binding by the output neurons. This is because the neuronal activity variables, that is the trace 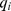 and firing rate 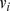, used in the two learning rules would reflect more recent neuronal activity and be less able to retain a memory of previous activity. This should lead to temporal binding over a shorter time window, and therefore retard the ability of output neurons to bind together and represent temporally proximal input patterns, which was hypothesized to be required for the development of head-centered output neurons. Thus, reducing the time constants 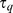 or 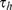 was expected to degrade the ability of the output layer to develop head-centered representations. The simulations otherwise had the same model parameters as the first experiment.

The two sets of simulations also aimed to investigate the relative efficacies of the trace learning rule 5 and hebbian learning rule 16 as mechanisms for temporal binding and consequently driving the development of head-centered output representations.

**Figure 9:**
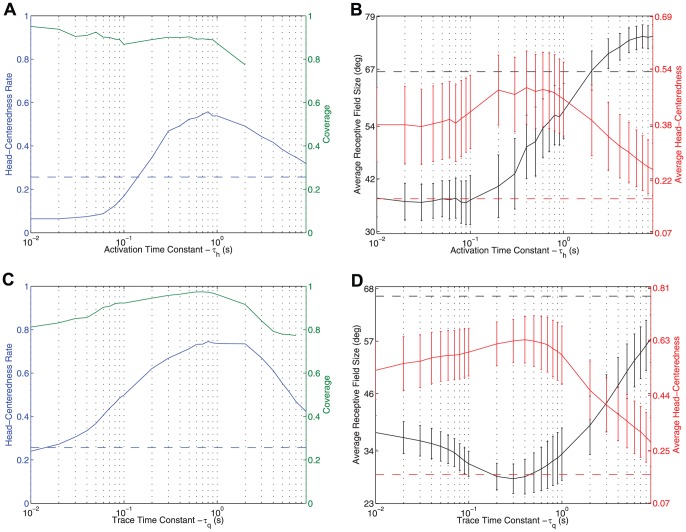
Varying time constants 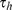 and 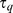. Simulations exploring the effects of varying the length of the activation time constants 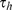 and the trace time constant 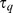 in the hebbian learning rule 16 and trace learning rule 5, respectively. The top row shows the series of simulations where a hebbian learning rule was used and the activation time constant was varied. The bottom row shows the series of simulations where a trace rule was used and the trace time constant was varied. The left plots show the fraction 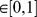 of output neurons that were deemed to be head centered (blue curve), and the coverage of the head centered training locations by the output neuron population (green curve). The right plots present the average size of the head centered receptive field among head-centered neurons (black curve), and the average head-centeredness value among all head-centered neurons (red curve). The error bars on these last two curves represent the standard deviations. The dashed line in each plot shows the corresponding quanitity in the untrained model, and since there was no coverage in the untrained model this line is absent.

Figure 9 shows the effects of varying the length of the activation time constant 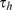 and the trace time constant 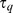 in the hebbian learning rule 16 and trace learning rule 5, respectively. The impact of varying the relevant time constant on the characteristics of the model was investigated by plotting key summary statistics as a function of the given time constant.

The observations from the simulations with the hebbian learning rule were as follows. For time constant 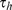 greater than 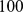
ms, the head-centeredness rate was above the baseline rate in the untrained model, which was 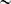
25%. The head-centeredness rate reached a maximum value of 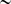
55% at 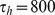
ms. For 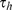 less than 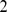
s, the coverage did not drop below 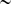
0.77. There was no coverage for the untrained model. The average head-centeredness among head-centered neurons remained above the untrained average of 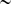
17% across the entire range of 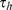, peaking at 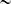
52% when 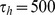
ms. Lastly, the average receptive field size among head-centered neurons was below the untrained model average of 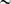
65 for all 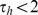
s. In summary, this showed that across a wide range of values of the activation time constant 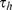 the prevalence of head-centered neurons and the compatability of their responses with a head-centered frame of reference were increased as a result of training. Although, for extreme values of 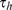 in either direction the model performed worse.

The results of the simulations with a trace learning rule were qualitatively similar. For simulations with the trace time constant 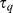 greater than 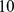
ms the head-centeredness rate was above the untrained rate. The head centeredness rate reached a maximum value of 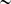
75% when 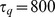
ms. There was coverage for all values of 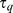 less than 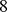
s, and the coverage peaked at 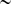
0.97 when 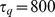
ms. The average head-centeredness among head-centered neurons remained above the untrained average across the entire range of values of 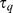, peaking at 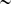
69% for 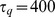
ms. The average receptive field size among head-centered neurons was below the untrained model average for all values of 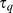. Overall, these results demonstrated that, across a broad range of values of 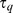, training led to an increase in the number of head-centered neurons and the compatability of their responses with a head-centered frame of reference. Moreover, the simulations with the trace learning rule produced somewhat better performance in terms of both head-centeredness rate and average head-centeredness than the simulations with the hebbian learning rule.

### Movement Statistics of Eyes, Head and Visual Targets

It was hypothesised that the model required that for some periods of time the visual target remained remained stationary in head-centered space while the eyes moved in order for output neurons to develop head-centered responses using the trace learning rule. This would cause different input patterns corresponding to a single head-centered target location to be clustered together in time. However, if the number of eye fixation positions 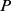 within each such period was reduced, then the trace learning rule would be prevented from binding together input patterns corresponding to the same head centered target locations. This would degrade the ability of the output layer of the model to form head-centered representations.

This experiment investigated the necessity of saccading between a sufficiently large number of successive eye position fixations for each fixed head-centered target location during training. It was expected that the output neurons would develop more strongly head-centered responses as the number of eye fixation positions 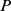 for each head centered target location was increased. More importantly, it was anticipated that the trained model would fail to produce output neurons with head-centered responses, in comparison to the untrained network, as 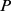 was reduced to 1. The sequence length was varied from 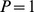 to 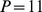, and the simulations otherwise had the same parameters as the first experiment.

**Figure 10:**
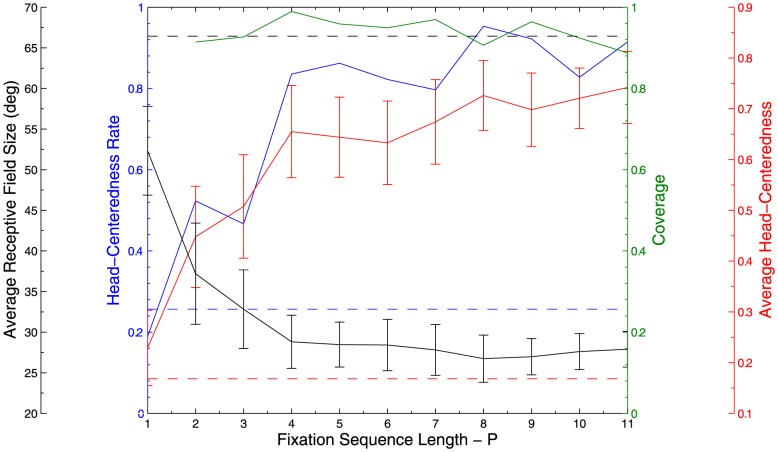
Varying fixation sequence length 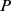. Simulations exploring the effects of varying the number, 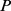, of eye fixation positions for each fixed head centered location of the visual target during training. Results are presented showing the response characteristics of the output neurons after 20 epochs of training. The dashed lines represent the corresponding values for the untrained network.

The effects of varying the length of the fixation sequence for each fixed head centered target location on the peformance of the trained model was investigated by plotting key summary statistics as a function of 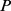 in Figure 10. Most importantly, the head-centeredness rate and the average head-centeredness increased almost monotonically with the length of the fixation sequence. Moreover, for all 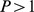 the head-centeredness rate and average head-centeredness were greater than the corresponding values for the untrained model, which were 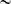
26% and 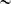
0.17, respectively. For all 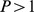, there was always coverage, which was never less than 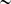
0.89, and which remained stable for all fixation sequence lengths. Like previous experiments, the average receptive field size among head-centered neurons decreased as head-centeredness rates increased, and remained stable for large values of 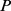.

These simulations confirmed that the output neurons developed more head centered responses as the length, 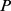, of the fixation sequence for each fixed head centered target location was increased. For the the shortest possible fixation sequence 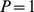, the output neurons in the trained model had a lower value of head centeredness rate than the untrained model. These observations confirmed that for output neurons to develop head centered responses, the eye position must move through a sufficiently large number, 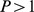, of successive fixations while the visual target remains in a fixed head-centered location.

## Discussion

This paper investigated the feasibility of the hypothesis that a biologically plausible process of visually-guided learning could produce neurons with head-centered receptive fields in a model that combined the following four core components: (i) input neurons that encoded the retinotopic location of a visual target and eye position through coupled receptive fields; (ii) output neurons that competed with each other through mutual inhibitory interactions; (iii) a synaptic learning rule that incorporated a memory trace of recent neural activity; and (iv) periods of time when the head and visual targets remained stationary while the eyes moved. It was successfully established, through computer simulation, that the combination of these core model components did allow for the development of head-centered visual representations in the output layer of the network. After training, output neurons were found to have receptive fields well aligned with the head-centered target locations the model was exposed to during training, and their receptive fields were stable across different eye fixation positions as required. Control experiments were then conducted to investigate whether the four core model components described above, having been established as sufficient, were also individually necessary for the model to successfully self-organize head-centered output representations.

It was shown that decoupling the visual and eye position dimensions of the receptive fields of input neurons prevented the model from developing head-centered output neurons during training. This result confirmed that input neurons with coupled visual and eye position receptive fields were indeed a necessary model component. Simply having the required input information, namely retinal target location and eye position, in a decoupled representation did not enable successful self-organization. This was because with input neurons that have decoupled receptive fields there does not exist any set of synaptic weights that can effect a mapping to head-centered output neurons. So no self-organisational synaptic learning process can solve this problem because there is in fact no solution. This can be understood by considering the following. For an output neuron to respond to a particular head centered location, it must respond to a set of many specific combinations of retinal target location and eye position that correspond to that head-centered location. These combinations will together cover large portions of the retinal target location space and eye position space. However, the inputs from these two spaces are represented independently. This means that the output neuron must have strengthened synaptic connections from input neurons representing a broad region of the retinal target location space, as well as input neurons representing a broad region of the eye position space. Indeed, all retinal target locations will map onto most head-centered locations (depending on eye position), and all eye positions will map most head-centered locations (depending on retinal target location). In this case, the output neuron will receive equal stimulation from the many possible combinations that can be constructed from these large portions of the retinal target location and eye position spaces, not just combinations corresponding to one specific head-centered location. In this case, the output neuron cannot respond selectively to just one head-centered location. Thus, with input neurons with decoupled receptive fields, there is no set of synaptic weights that can effect a selective mapping to head-centered output neurons. These results may therefore help to explain the functional significance of neurons with coupled visual and eye position receptive fields that have been reported in cortical area PO by [9]. This, in turn, would highlight the need for multiple stages of neural processing of the visual and eye position signals in the brain in order to develop neurons at an intermediate stage with coupled receptive fields, which then provide the required inputs for the model architectures presented. These neurons with coupled visual and eye position receptive fields may then fascilitate subsequent stages of neural processing, such as competitive trace learning to produce head-centered output representations.

The general form of network architecture used to develop head-centered output neurons is known as a *competitive neural network* [15], [20]. Such a network implements competitive interactions between the output neurons during training and testing. The competition is needed to encourage individual output neurons to learn to respond selectively to particular subsets of input patterns, with different output neurons responding to different subsets of input patterns. The subsets (categories) of input patterns that the output neurons learn to represent depend on the structure of the space of input patterns, the temporal order in which the input patterns are presented during training, and the kind of learning rule used to modify the synaptic weights. The model simulations reported above confirmed that competitive interactions were needed within the output layer in order to force individual output neurons to learn to respond selectively to subsets of input patterns corresponding to particular head-centered locations. This result was consistent with standard theory of learning and self-organization in competitive neural networks [15], [20].

It was shown that diminishing the efficacy of the synaptic learning rule to bind temporally proximal input patterns undermined the ability of the model to develop head-centered output neurons during training. Both the trace learning rule 5 and the hebbian learning rule 16 were sensitive to their relevant time constant, namely the trace time constant 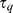 in equation 4 and activation time constant 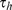 in equation 2, respectively. Only the middle range of values of these time constants allowed the model to develop head-centered output neurons. For very small time constants the memory trace of previous neural activity was dissipated too quickly for it to support learning of subsequent input patterns. For very large time constants the memory trace had too much inertia for previous neural activity to drive it up in the first place. Thus, in order to achieve effective temporal binding of input patterns, there was a need for a trace term that could be effectively driven up (i.e. a short enough time constant) but also remain for some period of time to support learning (i.e. a long enough time constant).

The fact that a Hebbian learning rule could effect temporal binding of input patterns was an important result. Previous research had appeared to show that a hebbian learning rule could not effect such temporal binding [23], [27]. However, this previous research had tested a more usual discrete time hebbian learning rule of the form 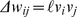. Such a discrete time version of a hebbian learning rule does not contain any memory trace of previous neural activity and so cannot perform temporal binding of input patterns. However, the hebbian learning rule 16 and activation equation 2 implemented in the simulations reported were time-continuous differential formulations. The differential equation 2 simulated neuronal activations with an exponential decay governed by the time constant 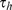, which ensured the activation 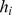 effectively represented a memory trace of recent neural activity. This memory trace of activity was then incorporated into the hebbian learning rule 16, which allowed the learning rule to perform temporal binding. An upshot of this result is that temporal binding in the brain may be performed by a simple hebbian learning rule without the need to invoke and explain additional mechanisms required for the explicit trace term 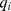 used in the trace learning rule 5. Indeed, temporal binding has recently been demonstrated in a more biophysically detailed model with spiking neurons and a STDP learning rule [29]. In these simulations, temporal binding was enhanced by increasing the time constant of the synaptic conductances, which controlled the flow of current into the postsynaptic neuron. These simulations provided one biologically plausible mechanism for temporal binding in the brain.

However, the simulations reported showed that the trace learning rule was more efficacious than the hebbian learning rule in terms of producing head-centered output neurons. In particular, simulations with the trace learning rule gave larger values for both the head-centeredness rate and average head-centeredness. One factor that might have contributed to this observation is that the trace learning rule allows postsynaptic neurons to learn regardless of whether they win the competition at the current time, while the hebbian learning rule requires the output neuron to win the competition at the current time to allow learning. Another factor responsible for the different efficacies of the two learning rules may be as follows. The pre- and postsynaptic terms in the hebbian learning rule represent the activities of these neurons over the same short time interval, which the rule associates together. Thus, there is only limited association and binding across time. In contrast, the pre- and postsynaptic terms in the trace learning rule represent the activities of these neurons over different, albeit nearby, time intervals. This helps to promote temporal binding of input patterns separated across time. These results suggest that it may be possible to further enhance the efficacy of the trace learning rule by incorporating an explicit time delay into the trace term. That is, the ability of the model to develop head-centered output neurons by temporal binding may be further improved by using a trace learning rule of the form 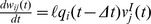 where 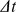 is a short delay of the order of, say, tens of milliseconds. The precise form of the trace learning rule and how this affects the performance of the model will remain an important issue for future research.

A core requirement for the model to produce head-centered output neurons through visually-guided learning is that there are periods of time during which the head and visual target remained fixed while the eyes move around a visual scene. This is a reasonable assumption because most visual stimuli remain static in the visual world for most of the time, and a primate will more frequently adjust its direction of gaze by moving its eyes rather than its head [19]. This will ensure that input patterns corresponding to a fixed head-centered location of the visual target will be clustered together in time. In this case, the trace learning rule is able to encourage individual output neurons to learn to respond selectively to subsets of input patterns corresponding to particular head-centered target locations. The simulations reported above confirmed the feasibility of this hypothesized mechanism for the development of head-centered visual representations in the primate dorsal visual pathway. Moreover, if these movement statistics were altered during training by reducing the number of eye fixation positions 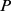 for each fixed head-centered target location, then, consistent with the hypothesis, this prevented the model from forming head-centered output representations. Of course, across different times, primates will experience a variety of different kinds of movement statistics of the eyes, head and visual targets. For example, sometimes a primate will move its head with respect to the static visual world, or a visual object will move while the eyes and head remain stationary. These are not the kind of movements required to build head-centered visual representations by binding of temporally proximal input patterns. However, this should not be a problem for the model, which should be capable of learning multiple different kinds of output representations. That is, when the eyes are moving while the head and visual target remain fixed, then some output neurons will learn to represent the head-centered location of the target. At other times, when the model is trained on different movement statistics, other output neurons may learn different kinds of representations such as eye-centered target locations. Indeed, evidence for this is provided by the fact that individual cortical regions such as LIP and PO do indeed contain a heterogenous population of neurons with different response characteristics, including both eye-centered and head-centered responses [11], [13].

The majority of past experimental and theoretical work in coordinate transformation from an eye- to a head-centered reference frame has focused on parietal areas LIP and 7a. In these areas eye position gain modulated retinotopic neurons are easily isolated, and consequently the planar eye position gain fields in these areas were studied in detail [8]. Later work identified eye position gain fields in area PO that were peaked rather than planar. Subsequent theoretical work demonstrated that this form of gain modulation improved the efficiency of the model by reducing the number of neurons necessary to encode the visual target position [10], and such modulation has also been used in other influential sensorimotor work with head-centered output representations [30], [31]. Some researchers speculated in early work that the difficulty in identifying head-centered neural representations at the single neuron level was evidence that eye position gain modulated neurons in parietal areas were the last stage of sensorimotor integration of signals representing the retinal location of visual targets and eye position, and that head-centered representations were only available at the population level of these eye position gain field neurons [8]. Subsequent experimental work did, however, reveal that multiple parietal areas, including PO, LIP and VIP, did have head-centered representations [11]–[13]. However, it still remains to identify the flow of signals between the various relevant cortical areas, and it is possible that experimental studies will not show an obvious, simple progression from eye-position gain modulated retinotopic neurons to head-centered neurons between two successive visual areas in the brain. For example, neuroanatomical studies have shown that area PO and area LIP are reciprocally connected [32], hence head-centered neurons in area LIP might develop using the trace learning principles described in this paper applied to the afferent synaptic connections received from eye-position gain modulated retinotopic neurons present in area PO. However, these head-centered neurons in LIP might then project back to other neurons in PO, which would then inherit head-centered response characteristics from the LIP inputs. This would give rise to a mixed population of both eye-position gain modulated retinotopic neurons and head-centered neurons in area PO, which is in fact what has been observed experimentally [11]. Nevertheless, the head-centered representations present in these areas would still initially develop by trace learning in the projections from eye-position gain modulated retinotopic neurons in area PO to area LIP. Another possible network architecture is that head-centered neurons in area PO develop by trace learning in the recurrent connections from eye-position gain modulated retinotopic neurons in area PO. Then, these head-centered neurons in area PO may project to neurons in area LIP that would then inherit these head-centered firing characteristics. There are many possible network architectures in which head-centered representations may develop without a readily apparant, simple progression from eye-position gain modulated retinotopic neurons in one visual area to head-centered neurons in a succeeding area. These more complex network architectures are characterised by the presence of either recurrent connections between neurons within an area, or the presence of both feedforward and feedback connections between different areas. Furthermore, these types of synaptic connectivity are indeed typical architectural features of the cortex [20]. Nevertheless, the trace learning mechanisms described in this paper may still operate in these more complex architectures in the manner described.

In conclusion, the model presented here provides a biologically plausible explanation of the mechanisms underpinning the development of head-centered visual representations in the macaque cerebral cortex. The model is distinguished from other previously published work by its relatively high degree of plausibility. The model uses a biologically plausible neural network architecture and local synaptic learning rule. In particular, the synaptic connections self-organize through a biologicaly plausible process of unsupervised, visually-guided, competitive learning. Unsupervised learning means that no implausible artificial teaching signal is used to set the firing rates of the output neurons during training [15], [20]. The model also utilises the natural movements of the eyes and head observed in primates [19]. The plausible way in which the required synaptic connections are set up within the model contrasts sharply with previously published work using error correction learning.

## Supporting Information

Appendix S1
**Eye-Centeredness Reference Frame Analysis.** This appendix demonstrates how the terms 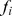 and 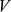, used to compute eye-centeredness, are derived.(PDF)

Appendix S2 **Head-Centered Receptive Field Location.** This appendix demonstrates how the head-centered receptive field location is derived.
(PDF)
