# Supplementary material for: A Model of Self-Organizing Head-Centered Visual Responses in Primate Parietal Areas
Source: PLoS One. 2013 Dec 3;8(12):e81406. doi: 10.1371/journal.pone.0081406 (PMC3857835; doi:10.1371/journal.pone.0081406)
Supplement: Appendix S1 — Eye-Centeredness Reference Frame Analysis. This appendix demonstrates how the terms and , used to compute eye-centeredness, are derived. (PDF) [file pone.0081406.s001.pdf]

Appendix S1: Eye-Centeredness Reference Frame Analysis

During testing, the visual target was located in head-centered locations

$$t_j = t_1 + \Delta h(j-1) \tag{1}$$

for $j = 1, \ldots, T$, and while in each location it was observed from eye positions

$$e_i = e_1 + \Delta e(i-1) \tag{2}$$

for $i = 1, \ldots, E$. The eye position shift Δe was set to a multiple of the head centered target location shift Δh to cause resampling of the neuron's response at the same retinal location for different eye positions, thereby providing a resampling of the response in both head-centered and eye-centered space across multiple eye positions.

The set of head-centered locations $\{t_1, \ldots, t_T\}$ corresponded to retinal locations $R_i = \{t_1 - e_i, \ldots, t_T - e_i\}$ when the model was fixating eye position e_i, and from this it is clear that among retinal locations common to all eye positions, $t_1 - e_1$ was the first and $t_T - e_E$ was the last, that is

$$\begin{aligned} \min \bigcap_i R_i &= t_1 - e_1 \\ \max \bigcap_i R_i &= t_T - e_E \end{aligned} \tag{3}$$

Therefore f_i and l_i, denoting the first and last position included from the ith response vector respectively, had to correspond to these two retinal locations respectively

$$t_{f_i} - e_i = t_1 - e_1 \tag{4}$$

$$t_{l_i} - e_i = t_T - e_E \tag{5}$$

We can resolve each equation to find an explicit formula for f_i and l_i in terms of i as follows.

By substituting equations 1 and 2 into equation 4 we obtain

$$(t_1 + \Delta h(f_i - 1)) - (e_1 + \Delta e(i-1)) = t_1 - e_1$$

Rearranging this gives

$$\begin{aligned} \Delta h(f_i - 1) - \Delta e(i-1) &= 0 \\ f_i &= \frac{\Delta e}{\Delta h}(i-1) + 1 \end{aligned} \tag{6}$$

By substituting equations 1 and 2 into equation 5 we obtain

$$(t_1 + \Delta h(l_i - 1)) - (e_1 + \Delta e(i-1)) = (t_1 + \Delta h(T-1)) - (e_1 + \Delta e(E-1))$$

Rearranging this gives

$$\begin{aligned} \Delta h(l_i - 1) - (e_1 + \Delta e(i-1)) &= \Delta h(T-1) - (e_1 + \Delta e(E-1)) \\ \Delta h(l_i - 1) - \Delta e(i-1) &= \Delta h(T-1) - \Delta e(E-1) \\ \Delta h(l_i - 1 - (T-1)) &= -\Delta e(E - 1 + (i-1)) \\ \Delta h(l_i - T) &= -\Delta e(E+i) \\ l_i &= T - \frac{\Delta e}{\Delta h}(E+i) \end{aligned} \tag{7}$$

We can also deduce the length V of the portion of each response vector that is used in the eye centered correlation analysis as follows. By definition, for each response vector

$$V = l_i - f_i + 1$$

Substituting in equations 6 and 7 gives

$$V = T - \frac{\Delta e}{\Delta h}(E + i) - \left(\frac{\Delta e}{\Delta h}(i - 1) + 1\right) + 1$$

Rearranging gives

$$\begin{aligned} V &= T - \frac{\Delta e}{\Delta h}(E + i) - \frac{\Delta e}{\Delta h}(i - 1) \\ &= T - \frac{\Delta e}{\Delta h}(E + i - (i - 1)) \\ &= T - \frac{\Delta e}{\Delta h}(E + 1) \qquad (8) \end{aligned}$$
