# Supplementary material for: A Model of Self-Organizing Head-Centered Visual Responses in Primate Parietal Areas
Source: PLoS One. 2013 Dec 3;8(12):e81406. doi: 10.1371/journal.pone.0081406 (PMC3857835; doi:10.1371/journal.pone.0081406)
Supplement: Appendix S2 — Head-Centered Receptive Field Location. This appendix demonstrates how the head-centered receptive field location is derived. (PDF) [file pone.0081406.s002.pdf]

Appendix S2: Head-Centered Receptive Field Location

For each output neuron, the head-centered receptive field location $\hat{h}_i$ for the ith eye position was calculated using the center of mass of the head-centered response vector at this eye position, that is

$$\hat{h}_i = \frac{\sum_{j=1}^{T} t_j \mathbf{R}[i,j]}{\sum_{j=1}^{T} \mathbf{R}[i,j]} \tag{1}$$

The values $\hat{h}_1, \ldots, \hat{h}_E$ may be regarded as estimates of a single global head-centered receptive field location h across all eye positions. In this case, the error $E(h)$ between the set of estimates $\hat{h}_1, \ldots, \hat{h}_E$ and the head-centered receptive field location h was defined as

$$E(h) = \sum_{i=1}^{E} \left(\hat{h}_i - h\right)^2 \tag{2}$$

This error was minimized over $h \in \mathbb{R}$ by first finding the derivative of this function

$$\begin{aligned} \frac{dE}{dh} &= \sum_{i=1}^{E} \frac{d\left(\left(\hat{h}_i - h\right)^2\right)}{dh} \\ &= -2\sum_{i=1}^{E}\left(\hat{h}_i - h\right) \\ &= -2\left(\sum_{i=1}^{E}\hat{h}_i - Eh\right) \end{aligned} \tag{3}$$

and then finding the critical points h^* by solving $\frac{dE}{dh}(h^*) = 0$

$$\begin{aligned} 0 &= -2\left(\sum_{i=1}^{E}\hat{h}_i - Eh^*\right) \\ 0 &= \sum_{i=1}^{E}\hat{h}_i - Eh^* \\ Eh^* &= \sum_{i=1}^{E}\hat{h}_i \\ h^* &= \frac{1}{E}\sum_{i=1}^{E}\hat{h}_i \end{aligned} \tag{4}$$

The fact that $\frac{dE(h_1)}{dh} < 0 < \frac{dE(h_2)}{dh}$ when $h_1 < h^* < h_2$ shows that h^* minimizes E. Therefore the head centred receptive field location of a given neuron was given by

$$\frac{1}{E}\sum_{i=1}^{E}\frac{\displaystyle\sum_{j=1}^{T} t_j \mathbf{R}[i,j]}{\displaystyle\sum_{j=1}^{T}\mathbf{R}[i,j]} \tag{5}$$
